# Comparison of prognosis after partial and total surgical resection for parathyroid carcinoma: an inverse probability of treatment weighting analysis of the SEER database

**DOI:** 10.3389/fendo.2023.1167508

**Published:** 2023-10-16

**Authors:** Shuai Jin, William C. Cho, Jiaxi Yang, Kaide Xia, Changxi Zhou

**Affiliations:** ^1^ School of Biology and Engineering (School of Health and Medicine Modern Industry), Guizhou Medical University, Guiyang, China; ^2^ Department of Clinical Oncology, Queen Elizabeth Hospital, Hong Kong, Hong Kong SAR, China; ^3^ Guiyang Maternal and Child Health Care Hospital, Guiyang Children’s Hospital, Guiyang, China; ^4^ National Clinical Research Center for Geriatric Diseases, People’s Liberation Army General Hospital, Beijing, China

**Keywords:** parathyroid carcinoma, partial surgical resection, total surgical resection, SEER database, inverse probability of treatment weighting

## Abstract

**Background:**

Complete resection of the tumor and the ipsilateral thyroid lobe at the primary surgery is the “gold standard” for the treatment of parathyroid carcinoma (PC). However, differences in the overall survival (OS) of patients with PC who underwent partial and total surgical resection remain to be determined.

**Methods:**

Data on patients with PC who underwent partial and total surgical resection were extracted from the Surveillance, Epidemiology and End Results (SEER) database (2000–2018). The X-tile software (https://medicine.yale.edu/lab/rimm/research/software/) was used to define the optimal cut-off values for continuous variables. The inverse probability of treatment weighting (IPTW) method was used to reduce the selection bias. IPTW-adjusted Kaplan–Meier curves and Cox proportional hazards models were used to compare the OS of patients with PC in the partial and total surgical resection groups.

**Results:**

A total of 334 patients with PC were included in this study (183 and 151 in the partial and total surgical resection groups, respectively). The optimal cut-off values for age at diagnosis were 53 and 73 years, respectively, while that for tumor size was 34 mm. In both the Kaplan–Meier analysis and univariable Cox proportional hazards regression analysis before IPTW, the difference in OS between the partial and total surgical resection groups was not statistically significant (p>0.05). These findings were confirmed in the IPTW-adjusted Kaplan–Meier analysis and multivariate Cox proportional hazards regression analysis (p>0.05). Subgroup analysis revealed that total surgical resection was beneficial for OS only in the subgroup with unknown tumor size.

**Conclusion:**

There was no significant difference in the prognosis of patients who underwent partial and total surgical resection. This finding may provide a useful reference for the treatment of PC.

## Introduction

Parathyroid carcinoma (PC) is one of the rarest malignancies of the endocrine system. It accounts for 0.005% of all malignancies in this system, and only approximately 5% of all cases of primary hyperparathyroidism. Since 2001, the incidence of PC has remained relatively stable (i.e., 10–13 cases per 10 million individuals) (1). PC exhibits a similar frequency in both men and women, mostly developing between 45 and 59 years of age (2, 3). Germline heterozygous inactivating mutations of the CDC 73 oncogene account for approximately 50-75% of familial PCs; more than 75% of disseminated PCs have a double allele somatic inactivation/loss of CDC 73 (4). The cause of PC is unknown and may be related to genetic factors or hyperparathyroidism (5). Moreover, the diagnosis of PC is difficult owing to its clinical, radiological, and histological similarity to parathyroid adenoma (6). The rate of PC progression is slow; the rates of lymph node and distant metastasis are approximately 5% and 2%, respectively (7, 8). In previous reports, the 5- and 10-year survival rates of patients with PC were approximately 80% and 65%, respectively (9). Most deaths among those patients are attributed to uncontrolled hypercalcemia rather than target organ damage due to tumor load (10). Thus far, a prognostic staging system for patients with PC (similar to the TNM staging system) has not been developed, and there is no consensus regarding the treatment of PC (11).

Surgery is an important predictor of PC prognosis and the only curative treatment for this disease (12). Previous studies on PC recommended radical surgery with microscopic negative margins (13). Total resection should include the tumor, as well as the ipsilateral well-defined thyroid lobes and adjacent involved structures. Lymph nodes in the tracheoesophagus, paratracheal, and upper mediastinum should also be removed (14). Limited parathyroidectomy is associated with a significantly worse prognosis than extensive tumor resection (15–17). However, a lack of significant correlation between radical resection and improved survival has been observed in other studies (13, 18).

Owing to the rarity of PC and the lack of prospective studies, investigations based on small samples produce conflicting results. The prognostic impact of the extent of surgery on patients with early-stage PC is unclear. Therefore, our study was designed to assess the difference in the long-term prognosis of patients with early-stage PC who underwent partial and total surgical resection. For this purpose, we used data from the Surveillance, Epidemiology, and End Results (SEER) database. Inverse probability of treatment weighting (IPTW) was used to reduce the selection bias.

## Materials and methods

### Data source and patient selection

This study cohort was retrieved from the SEER program; this database records patient data from 18 population-based cancer registries, covering approximately 27.8% of the population of the United States of America (19). The SEER registries collect data on the demographics, tumor characteristics, stage at diagnosis, primary tumor site, initial course of treatment, and follow-up status of patients with cancer. The data used in this study are publicly available, plus version data from April 2021, containing information on treatment for the period 2000–2018. All data were downloaded through the SEER*Stat software (Version 8.4.0, https://seer.cancer.gov/seerstat/). SEER is an open-access public database, and private patient information has been officially withheld from this database. Hence, approval from an institutional review board was not required.

**Figure 1:**
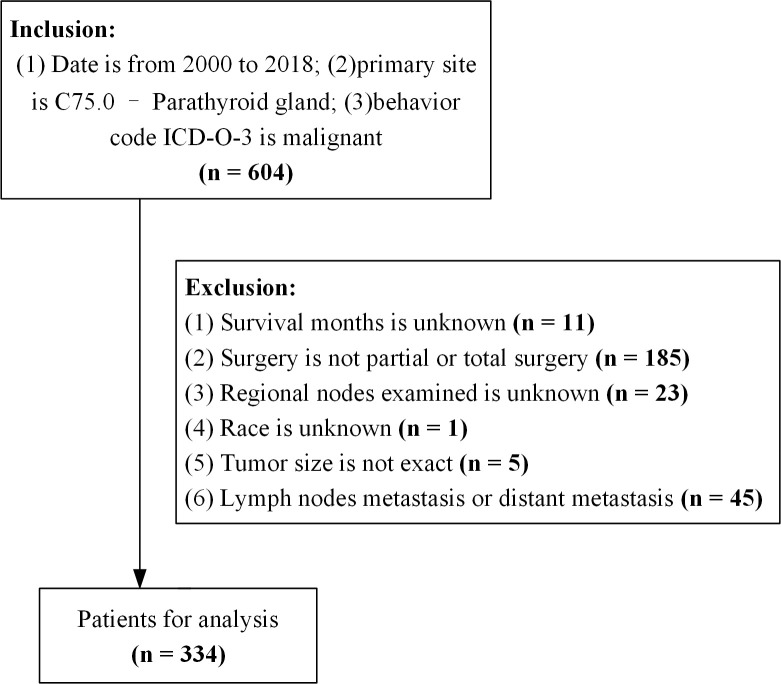
Flowchart of the data process. ICD-O-3, International Classification of Disease for Oncology version 3.

The criteria for data extraction were as follows (1): date from 2000 to 2018; (2) primary tumor site was C75.0 - parathyroid gland; and (3) diagnosis of parathyroid cancer by experienced pathologists. We extracted patient demographic variables (age at diagnosis, sex, race, and marital status), tumor characteristics (tumor size, number of malignant tumors, lymph node status, and distant metastases), treatment status (regional lymph node dissection, radiotherapy, and surgical modality), months of survival, and survival status. The exclusion criteria were: (1) unknown duration of survival; (2) surgical procedures other than partial and total resection; (3) presence of lymph node metastases; (4) lymph nodes and distant metastases at the time of diagnosis; and (5) incomplete information (**Figure 1**).

### Variable recode

Variable reassignment was not performed for sex and race in the extracted data. Age at diagnosis and tumor size were converted to categorical variables after determining the cut-off values using the X-tile software (https://medicine.yale.edu/lab/rimm/research/software/). Marital status was categorized as “married at diagnosis” and “other”, the number of malignancies was categorized as “single” and “multiple”, radiotherapy was categorized as “yes” and “no/unknown”, and regional nodes examined were categorized as “yes” or “no”; the above variables were binary processed. According to the SEER Program Surgery Codes Manual, surgical procedure codes 30 and 40 denote partial and total surgical resection. In addition, the patient profiles were screened for lymph nodes and distant metastases based on variables, such as the extent of disease for regional nodes. According to the explanation of variables in the SEER database, overall survival (OS) was the endpoint of interest in this study. OS was defined as the period from the date of PC diagnosis to that of death from any cause.

### Statistical analysis

Categorical variables are shown as frequencies and their proportions. The baseline characteristics of patients before and after matching were assessed using standard mean difference (SMD); a value >0.1 indicated that the information was unbalanced between the groups (20). Adjustment of observed differences in baseline covariates between the two intervention groups was performed using the IPTW method to eliminate selection bias (21). Univariate analysis of the impact of patient characteristics on OS was performed using the Kaplan–Meier method; the log rank method (Mantel–Cox) was utilized to assess statistical significance. Variables with a p-value <0.2 in the univariate analysis were included in a multivariate Cox proportional hazards regression model to investigate the effect of both surgical procedures on OS. Statistical analyses and data visualization were performed using R (https://www.r-project.org/, version 4.2.1) in the RStudio environment (https://posit.co/download/rstudio-desktop/, version 2022.12.0 + 353). All statistical analyses involved two-sided testing, and p-values <0.05 denoted statistically significant differences.

## Results

### Patient characteristics

A total of 334 PC patients met the inclusion criteria with a median follow-up time of 205 months. Of those, 183 and 151 patients underwent partial and total surgical resection, respectively. After processing, 53 and 73 years were identified as the best cut-off values for age at diagnosis, while 34 mm was the best cut-off value for tumor size. Of the patients, 53.6% were male, 36% were aged <53 years, 51% were aged 53–73 years, and 62% were married at the time of diagnosis. Moreover, 50% of patients had a tumor size <34 mm, and 74% had a single primary malignancy. Radiotherapy and regional lymph node testing were performed in 10% and 34% of the patients, respectively. In the unweighted data, the SMDs of age at diagnosis, sex, race, tumor size, and radiotherapy were >0.1, thereby denoting bias in the original data. After IPTW adjustment, the SMDs of all variables were <0.1, indicating that the partial and total surgical resection groups were comparable (**Table 1**).

**Table 1:** Selected baseline characteristics of patients who underwent partial and total surgical resection before and after IPTW.

Characteristic	Unweighted study population n (%)			Weighted study population n (%)		
	Partial surgical resection (n=183)	Total surgical resection (n=151)	SMD	Partial surgical resection	Total surgical resection	SMD
**Age at diagnosis**			0.171			0.006
<53 years	66 (36.1)	43 (28.5)		110.5 (33.1)	111.5 (33.4)	
53–73 years	94 (51.4)	84 (55.6)		176.1 (52.7)	175.5 (52.5)	
>73 years	23 (12.6)	24 (15.9)		47.3 (14.2)	47.2 (14.1)	
**Sex**			0.131			0.002
Female	85 (46.4)	80 (53.0)		164.2 (49.2)	164.0 (49.0)	
Male	98 (53.6)	71 (47.0)		169.7 (50.8)	170.3 (51.0)	
**Race**			0.186			0.005
Black	27 (14.8)	31 (20.5)		59.7 (17.9)	59.5 (17.8)	
Other	17 (9.3)	9 (6.0)		25.8 (7.7)	25.5 (7.6)	
White	139 (76.0)	111 (73.5)		248.4 (74.4)	249.3 (74.6)	
**Tumor size**			0.119			0.005
1–34 mm	91 (49.7)	77 (51.0)		167.9 (50.3)	167.5 (50.1)	
>34 mm	36 (19.7)	35 (23.2)		69.1 (20.7)	69.9 (20.9)	
Unknown	56 (30.6)	39 (25.8)		97.0 (29.0)	97.0 (29.0)	
**Number of tumors**			0.080			0.006
Multiple	48 (26.2)	45 (29.8)		93.7 (28.1)	92.9 (27.8)	
Single	135 (73.8)	106 (70.2)		240.2 (71.9)	241.4 (72.2)	
**Marital status**			0.042			0.001
Married	114 (62.3)	91 (60.3)		205.6 (61.6)	206.1 (61.7)	
Other	69 (37.7)	60 (39.7)		128.3 (38.4)	128.2 (38.3)	
**Radiotherapy**			0.164			0.005
No/Unknown	165 (90.2)	128 (84.8)		291.9 (87.4)	292.9 (87.6)	
Yes	18 (9.8)	23 (15.2)		42.0 (12.6)	41.4 (12.4)	
**RNE**			0.040			0.007
No	121 (66.1)	97 (64.2)		216.8 (64.9)	216.0 (64.6)	
Yes	62 (33.9)	54 (35.8)		117.2 (35.1)	118.4 (35.4)	

IPTW, inverse probability of treatment propensity-score weighting; RNE, regional nodes examined, SMD, standard mean difference.

### Survival analyses

**Figure 2:**
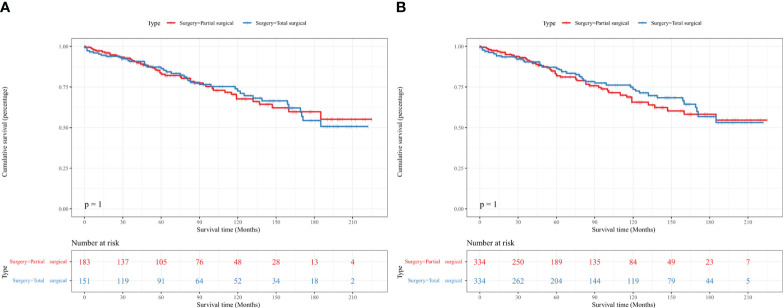
Kaplan–Meier survival curves showed no difference in the OS of patients with PC between the partial and total surgical resection groups before **(A)** and after **(B)** IPTW. IPTW, inverse probability of treatment propensity-score weighting; OS, overall survival; PC, parathyroid carcinoma.

Kaplan–Meier analysis did not show a statistical difference in the OS of patients with PC between the partial and total surgical resection groups before IPTW (p=1.000) (**Figure 2A**). This result was confirmed in the IPTW-adjusted Kaplan–Meier analysis (p=1.000 in the IPTW-adjusted log-rank test) (**Figure 2B**). In the univariate Cox proportional hazards regression model without IPTW, there was no difference in OS between the two groups (p=0.997). The multivariate Cox proportional hazards regression model with raw data confirmed that age, tumor size, and marital status were independent prognostic factors for OS in patients with PC. Consistent with the results of the pre-IPTW model, the IPTW-adjusted univariate Cox proportional hazards regression model showed that the mode of surgery remained statistically non-significant (p=0.662), whereas age at diagnosis, tumor size, and marital status were independent prognostic factors in the corrected data (**Table 2**).

**Table 2:** Univariate and multivariate Cox regression analyses of OS in patients with PC before and after IPTW.

Characteristic	Unadjusted				IPTW-adjusted			
	Univariate analysis		Multivariate analysis		Univariate analysis		Multivariate analysis	
	HR (95% CI)	p-value	HR (95% CI)	p-value	HR (95% CI)	p-value	HR (95% CI)	p-value
Age at diagnosis								
<53 years	Reference		Reference		Reference		Reference	
53–73 years	4.55 (2.34–8.85)	<0.001	4.25 (2.15–8.41)	<0.001	1.95 (1.07–3.55)	0.028	2.24 (1.14–4.40)	0.020
>73 years	1.94 (1.10–3.41)	0.022	2.35 (1.32–4.18)	0.004	4.69 (2.39–9.19)	<0.001	4.29 (2.10–8.74)	<0.001
Sex								
Female	Reference							
Male	1.24 (0.80–1.90)	0.334			1.24 (0.80–1.92)	0.346		
Race								
Black	Reference		Reference		Reference		Reference	
Other	0.56 (0.24–1.31)	0.179	1.13 (0.46–2.78)	0.794	0.70 (0.28–1.74)	0.443	1.35 (0.52–3.53)	0.541
White	0.51 (0.31–0.83)	0.007	0.75 (0.44–1.27)	0.282	0.51 (0.30–0.85)	0.010	0.74 (0.43–1.27)	0.269
Tumor size								
1–34 mm	Reference		Reference		Reference		Reference	
>34 mm	1.95 (1.10–3.49)	0.023	1.82 (1.01–3.27)	0.046	1.97 (1.09–3.59)	0.026	1.87 (1.03–3.40)	0.038
Unknown	2.41 (1.47–3.96)	0.001	2.08 (1.25–3.45)	0.005	2.27 (1.37–3.77)	0.001	1.95 (1.17–3.25)	0.011
Number of tumors								
Multiple	Reference				Reference		Reference	
Single	0.77 (0.49–1.20)	0.250			0.68 (0.43–1.06)	0.085	0.89 (0.55–1.45)	0.643
Marital status								
Married	Reference		Reference		Reference		Reference	
Other	2.56 (1.66–3.95)	<0.001	2.43 (1.51–3.92)	<0.001	2.38 (1.52–3.71)	<0.001	2.28 (1.40–3.71)	<0.001
Type of surgery								
Partial surgical resection	Reference				Reference			
Total surgical resection	1.00 (0.65–1.53)	0.997			0.91 (0.59–1.41)	0.662		
Radiotherapy								
No/Unknown	Reference				Reference			
Yes	1.36 (0.72–2.57)	0.346			1.46 (0.80–2.66)	0.213		
RNE								
No	Reference				Reference			
Yes	0.99 (0.63–1.56)	0.966			0.94 (0.59–1.49)	0.781		

CI, confidence interval; HR, hazard ratio; IPTW, inverse probability of treatment propensity-score weighting; OS, overall survival; RNE, regional nodes examined.

### Subgroup analysis

**Figure 3:**
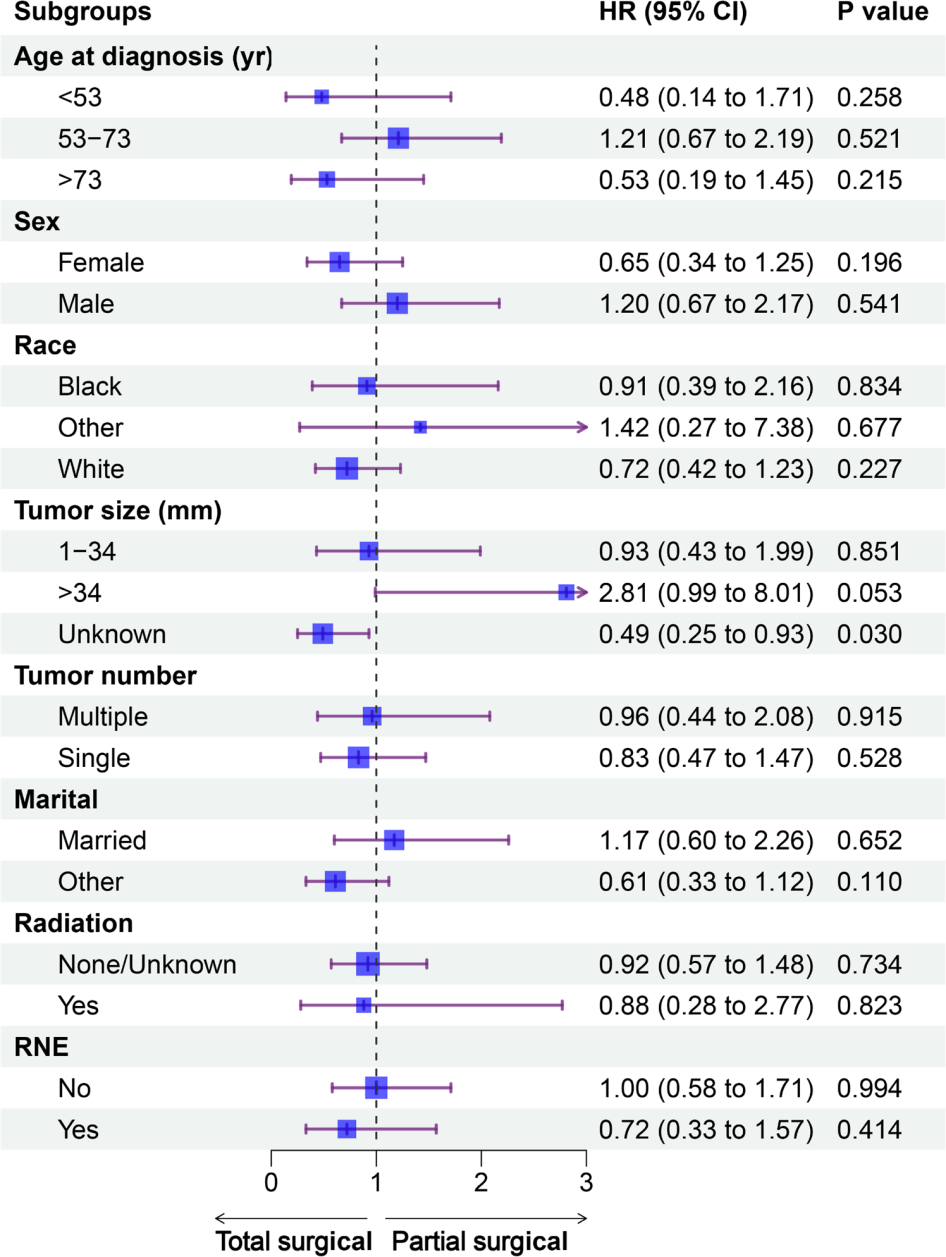
Forest plot of patients with PC included in the subgroup analysis (partial *vs*. total surgical resection). CI, confidence interval; HR, hazard ratio; PC, parathyroid carcinoma; RNE, regional nodes examined; yr, year.

The forest plot did not show a difference in prognosis between the partial and total surgical resection groups, except for the subgroup of unknown tumor size. In the group with unknown tumor size, total surgical resection was associated with better OS than partial surgery (hazard ratio: 0.49, p=0.030). In the subgroup with tumor size >34 mm, the p-value was 0.053, almost reaching the threshold of statistical significance. In the subgroup analysis of other variables, there was no difference in the prognosis for the partial and total surgical resection groups (**Figure 3**).

## Discussion

In this study, we sought to identify differences in the prognostic impact of partial and total surgical resection on the OS of patients with early-stage PC by analyzing data from the representative SEER database. Owing to the wide coverage of the SEER database and the relatively complete data collection, it is possible to investigate rare types of cancer, such as PC. To the best of our knowledge, this is the first study using the IPTW method to achieve the aforementioned objective.

Due to the rarity of PC, the available literature on this disease mostly comprises reviews and case reports. Nevertheless, several studies have investigated the prognostic factors of OS and cancer-specific survival in patients with PC, providing data to support the treatment and management (11, 22–25). Most of these studies involved database analyses, such as the SEER and the American College of Surgeons National Cancer Database. Remarkably, the number of patients with lymph nodes and distant metastases in these studies was very low (typically <10), which may have led to biased results. PC is characterized by relatively lazy features and progresses slowly. Thus, it is of great practical importance to investigate the prognosis of patients with early-stage disease in studies involving relatively large sample sizes. The results of the multivariate Cox proportional hazards regression model before and after IPTW were consistent, confirming that age at diagnosis and tumor size were prognostic factors for cancer-specific survival in patients with PC. These findings are consistent with those of previous studies (22, 26). Previous studies on the role of marital status as a prognostic factor affecting OS in patients with PC reported inconsistent conclusions (24, 26). This discrepancy in results may be due to differences in the criteria used in studies for the selection of patients. Hence, additional studies are warranted to validate the present result.

Surgery is an important factor in the prognosis of patients with PC. Compared with partial surgical resection, primary surgery involving total surgical resection provides the best chance of cure and is associated with a significantly better prognosis (12, 13). However, studies have shown a lack of significant correlation between total surgical resection and improved OS in patients with PC (18, 27). In this study, after adjusting for baseline data, we did not find a significant difference in the effect of partial and total surgical resection on the prognosis of OS in patients with PC. This result was consistent with previous findings (28, 29). Therefore, it is reasonable to suggest that total surgical resection is a better choice than partial surgical resection. This difference may be associated with the use of secondary and multiple surgeries. Studies have shown that extending the surgical resection and increasing the number of surgeries reduces the rates of disease recurrence and mortality in patients with PC (17, 30). Another possible explanation is that the surgical information included in the cancer registry does not adequately describe the extent of resection (3, 31, 32). In a previous study, prophylactic cervical lymph node dissection was recommended for patients with primary tumors measuring >3 cm (31). The subgroup analysis of this study found that total surgical resection did not achieve better results among patients with primary tumors measuring >34 mm. Considering the low incidence of metastasis in the PC region, prophylactic cervical lymphadenectomy should be performed with caution (33, 34). Parathyroid cancer is characterized by a high recurrence rate. Moreover, effective treatment options for this disease (beyond primary total surgical resection) are currently limited. Therefore, a high preoperative index of suspicion and regular aggressive review is essential to optimize patient care (35). In addition, multi-institutional studies with standardized surgical procedures are warranted to further assess differences between partial and total surgery.

Although we applied the IPTW method to correct the baseline features and obtained a relatively reliable conclusion. The limitations of this population-based database investigation should be acknowledged. Firstly, this study involved a retrospective analysis, which may have been biased by the removal of cases with incomplete information. Secondly, several important indicators that have been associated with PC (e.g., serum parathyroid hormone and calcium levels, and disease recurrence) are not included in the SEER database. Therefore, we were unable to examine the effects of such indicators on the prognosis of patients with PC in relation to the surgical approach. Thirdly, the SEER database does not provide PC gene information, especially the CDC73 mutation status. Again, due to the rarity of PC cases, even national databases do not include many cases (especially patients with more than 15 years of follow-up), which may lead to biased study results. Finally, clinical and oncology variables require precise coding for analysis. For example, the SEER definition of surgical modality is imprecise, and the “Surgical Codes Other Sites 2023” manual, which is available on the official website of the database, does not specifically address the coding of parathyroid surgery.

## Conclusion

In patients with early-stage PC, there was no significant difference in OS following partial and total surgical resection. Our findings may provide insights and would assist in the selection of the appropriate surgical approach for patients with PC.

## Data availability statement

The original contributions presented in the study are included in the article/supplementary material. Further inquiries can be directed to the corresponding authors.

## Ethics statement

Ethical review and approval were not required for the study on human participants in accordance with the local legislation and institutional requirements. Written informed consent from the participants or the participants’ legal guardian/next of kin was not required to participate in this study in accordance with the national legislation and the institutional requirements.

## Author contributions

SJ and CZ were responsible for the conception and design of the study. SJ also collected data and performed the statistical analysis. KX, WC and JY wrote and edited the manuscript. WC also made contributions to manuscript review and revision. CZ provided financial support. All authors contributed to the article and approved the submitted version.
